# Supplementary material for: MYBPC3 D389V Variant Induces Hypercontractility in Cardiac Organoids
Source: Cells. 2024 Nov 19;13(22):1913. doi: 10.3390/cells13221913 (PMC11592734; doi:10.3390/cells13221913)
Supplement: Supplementary file 1 [file cells-13-01913-s001.zip › R1 Online Supplemental Figures.pdf]

Online Supplemental Figures

MYBPC3 D389V Variant Induces Hypercontractility in Cardiac Organoids

Darshini Desai[a€α], Taejeong Song[a&], Rohit R. Singh[a§], Akhil Baby[a,b&], James McNamara[a,^], Lisa Green[a], Pooneh Nabavizadeh[a], Mark Ericksen[a], Sholeh Bazrafshan[a#], Sankar Natesan[b] and Sakthivel Sadayappan[a&*]

[a]Center for Cardiovascular Research, Division of Cardiovascular Health and Disease, Department of Internal Medicine, University of Cincinnati College of Medicine, Cincinnati, OH 45267, USA.

[b]Department of Genetic Engineering, School of Biotechnology, Madurai Kamaraj University, Madurai 625021, India.

[&]**Current Address**: Department of Cellular & Molecular Medicine, Sarver Heart Center, University of Arizona College of Medicine, Tucson, AZ 85724, USA

[α]**Current Address**: Icahn School of Medicine, Cardiovascular Research Institute, Mount Sinai, New York, USA.

[§]**Current Address**: Amgen Research, Department of Cardiometabolic Disorders, Amgen, South San Francisco, California, USA.

^Current Address: Murdoch Children's Research Institute, The Royal Children's Hospital, Parkville, Victoria 3052; Department of Physiology and Centre for Muscle Research, School of Biomedical Sciences, The University of Melbourne, Parkville, Victoria, 3010, Australia; Novo Nordisk Foundation Centre for Stem Cell Medicine (reNEW), Murdoch Children's Research Institute, Melbourne, Victoria, Australia.

[#]**Current Address**: Department of Pathology and Laboratory Medicine, University of Cincinnati Medical Center, Cincinnati, OH 45267, USA

Running title: Cardiac Organoids for HCM

[€]Correspondence to Darshini Desai, Ph.D., Icahn School of Medicine, Cardiovascular Research Institute, Mount Sinai, New York, NY. Phone: +1-513-356-1099; Email: davdesai21@gmail.com

*Co-correspondence to Sakthivel Sadayappan, Ph.D., MBA, Department of Cellular & Molecular Medicine, Sarver Heart Center, University of Arizona College of Medicine-Tucson, Tucson, AZ 85724-

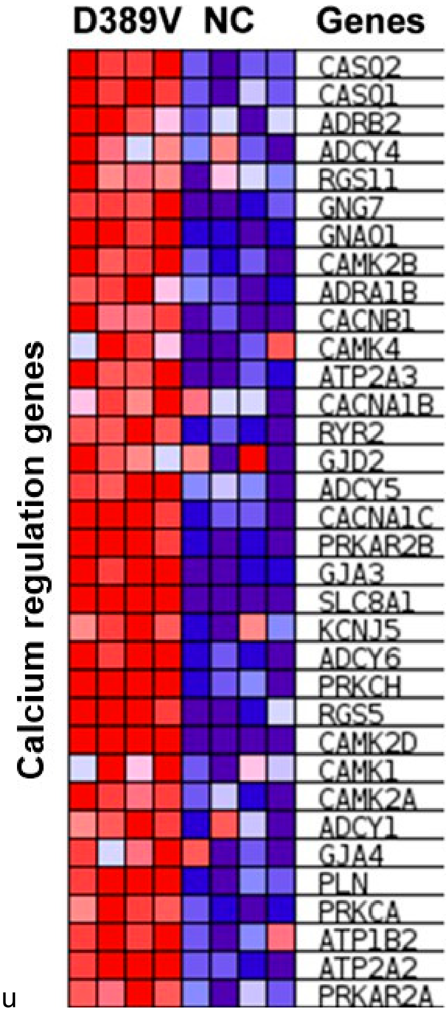

5217. Phone: +1-520-621-1583; Email: sadayappan@arizona.edu

Supplemental Figure S1: RNAseq analysis of calcium regulation pathway: Heatmap representation of differentially expressed transcripts in calcium regulation pathway identified from RNA-seq analysis of NC and D389V cardiac organoids. Red boxes (UP) represent significantly up-regulated genes, and blue boxes (DOWN) represent significantly down-regulated genes (Fold change cutoff 2.0 Adjusted p-value <0.05). n=4 sample sets, 25-30 organoids in each set.

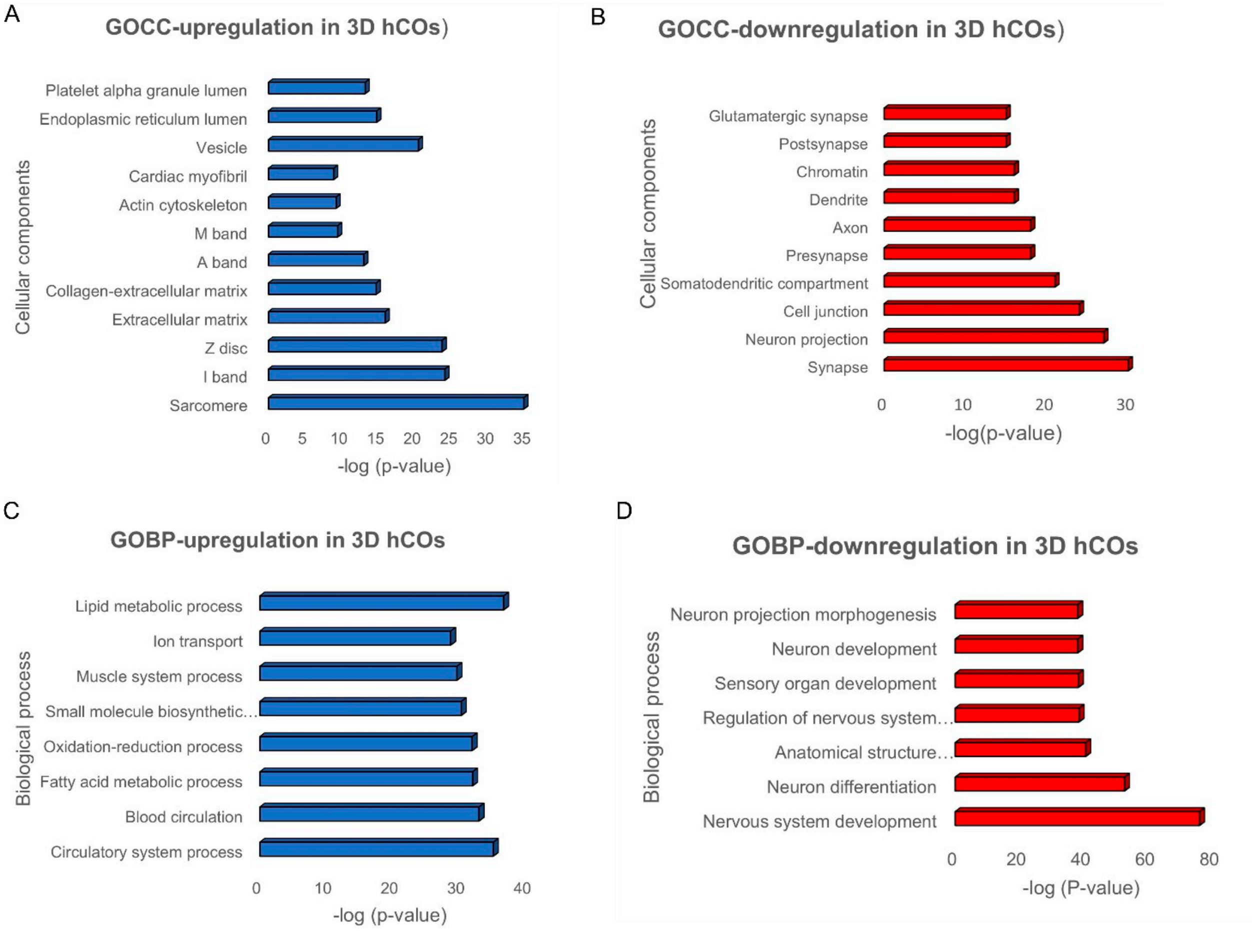

Supplemental Figure S2: RNA seq data analysis demonstrating comparison of upregulated and downregulated DEGs in 3D cardiac organoids compared to 2D cardiomyocytes. (**A**) Upregulation of gene expression of Gene ontology cellular components (GOCC) in 3D cardiac organoids compared to 2D cardiomyocytes. (**B**) Downregulation of gene expression of GOCC in 3D cardiac organoids compared to 2D cardiomyocytes. (**C**) Upregulation of gene expression of Gene ontology biological process (GOBP) in 3D cardiac organoids compared to 2D cardiomyocytes. (**D**) Downregulation of gene expression of GOBP in 3D cardiac organoids compared to 2D cardiomyocytes. Functional enrichment analysis of DEGs was generated using the iDEP 9.5 analysis tool. The length of the bar represents the -log10 (P-value) of each pathway; GO biological process and GO cellular component were shown on y-axis.

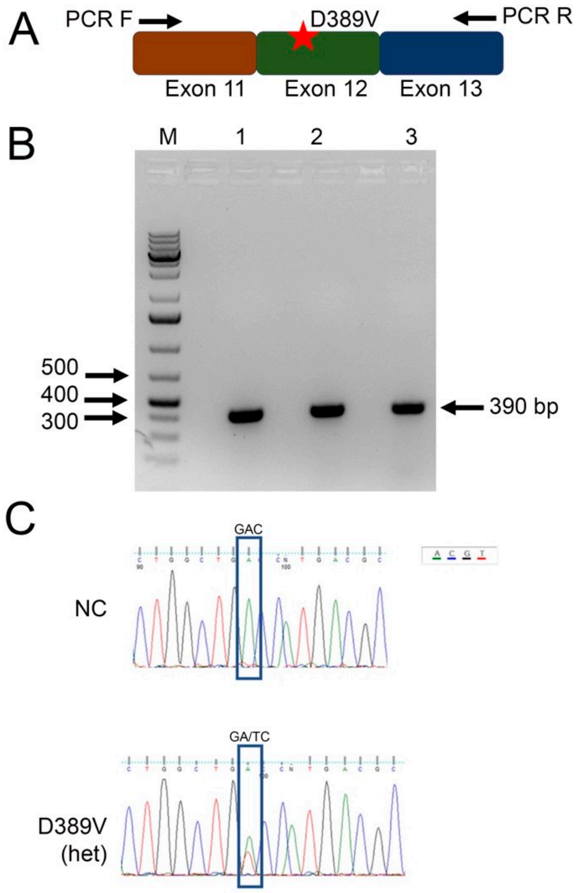

Supplemental Figure S3. Determination of mutant mRNA in D389V hiPSC-CMs. (**A**) A schematic diagram illustrating the location of the D389V variant in exon 12 and the directions of the polymerase chain reaction (PCR) using the primers (Forward and Reverse). (**B**) The PCR product of 390bp was run on the gel from the hiPSC-CMs derived from NC and D389V lines. Lanes 1 and 2 products were from D389V samples, and lane 3 product was from NC. (**C**) Sanger sequencing shows two peaks of A and T in D389V heterozygous (Het) samples, compared to the non-carrier (NC).

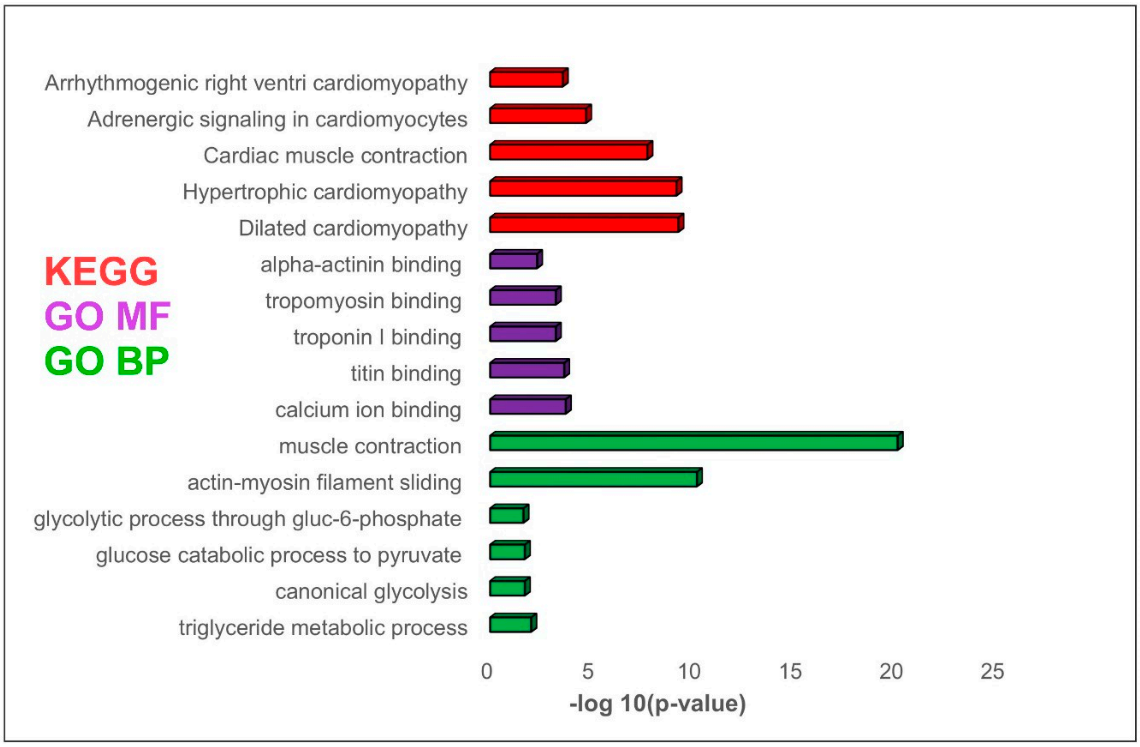

Supplemental Figure S4. RNA seq data analysis demonstrating a comparison of upregulated pathways in D389V hCOs compared to NC hCOs. Bar graphs showing upregulated KEGG (red), Gene ontology molecular function (GO MF, purple), and Gene ontology biological process (GO BP, green) pathways in D389V hCOs. The length of the bar represents the -log10 (P-value) of each pathway (Fold change cutoff 2.0 Adjusted p-value <0.05). n=4 sample sets, 25-30 organoids in each set.

49 Spadina Ave.
Suite 200 Toronto ON
M5V 2J1 Canada
www.biorender.com

Confirmation of Publication and Licensing Rights

May 27th, 2024
Science Suite Inc.

Subscription:	*Institution*
Agreement	*TF26VAUXQ*
number: Journal	*C*
name:	*bioRxiv*

To whom this may concern,

This document is to confirm that Sakthivel Sadayappan has been granted a license to use the BioRender content, including icons, templates and other original artwork, appearing in the attached completed graphic pursuant to BioRender's Academic License Terms. This license permits BioRender content to be sublicensed for use in journal publications.

All rights and ownership of BioRender content are reserved by BioRender. All completed graphics must be accompanied by the following citation: “Created with BioRender.com”.

BioRender content included in the completed graphic is not licensed for any commercial uses beyond publication in a journal. For any commercial use of this figure, users may, if allowed, recreate it in BioRender under an Industry BioRender Plan.

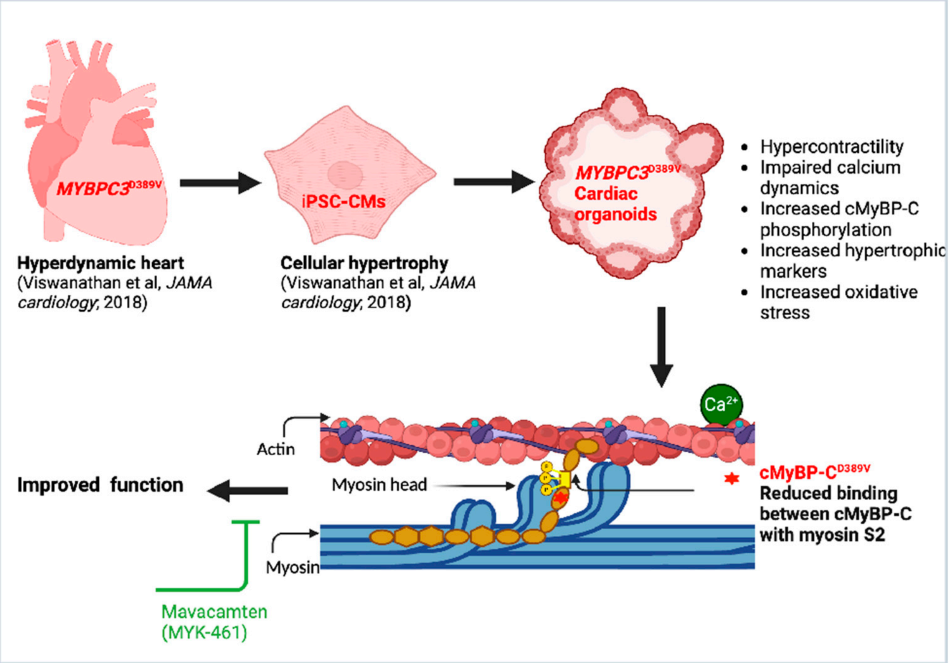

For any questions regarding this document, or other questions about publishing with BioRender refer to our BioRender Publication Guide, or contact BioRender Support at support@biorender.com
